# Sigma-1 Receptor Chaperone at the ER-Mitochondrion Interface Mediates the Mitochondrion-ER-Nucleus Signaling for Cellular Survival

**DOI:** 10.1371/journal.pone.0076941

**Published:** 2013-10-18

**Authors:** Tomohisa Mori, Teruo Hayashi, Eri Hayashi, Tsung-Ping Su

**Affiliations:** Cellular Pathobiology Section, Intramural Research Program/NIDA/NIH/DHHS, Baltimore, Maryland, United States of America; Sanford-Burnham Medical Research Institute, United States of America

## Abstract

The membrane of the endoplasmic reticulum (ER) of a cell forms contacts directly with mitochondria whereby the contact is referred to as the mitochondrion-associated ER membrane or the MAM. Here we found that the MAM regulates cellular survival via an MAM-residing ER chaperone the sigma-1 receptor (Sig-1R) in that the Sig-1R chaperones the ER stress sensor IRE1 to facilitate inter-organelle signaling for survival. IRE1 is found in this study to be enriched at the MAM in CHO cells. We found that IRE1 is stabilized at the MAM by Sig-1Rs when cells are under ER stress. Sig-1Rs stabilize IRE1 and thus allow for conformationally correct IRE1 to dimerize into the long-lasting, activated endonuclease. The IRE1 at the MAM also responds to reactive oxygen species derived from mitochondria. Therefore, the ER-mitochondrion interface serves as an important subcellular entity in the regulation of cellular survival by enhancing the stress-responding signaling between mitochondria, ER, and nucleus.

## Introduction

Traditionally, the ER has been classified into the smooth and rough ER. However, the ER is known to be composed of distinct substructures including the ER exit site and the mitochondria-associated ER membrane (MAM). The MAM is a subdomain of the ER that engages in a direct physical association with mitochondria [1]–[5]. The MAM integrates many signaling pathways and is important for cellular survival because it serves as the “tunnel” for lipid transport and Ca^2+^ signaling between the ER and mitochondria [2], [6], [7]. We previously found that Sig-1Rs, which relate to addictive processes and learning and memory [8], are ER chaperones that localize specifically at the MAM and regulate a variety of cellular functions including Ca^2+^ signaling between ER and mitochondria, neuronal differentiation, ion channel activities, and notably cellular survival [9]–[13]. However, exactly how Sig-1Rs regulate those various biological processes, particularly those involved in cellular survival at the MAM, is not fully clarified.

The lumen of the ER provides a unique environment for proper protein folding and assembly of newly synthesized proteins. Under normal physiological conditions, the protein level in the ER is maintained in a homeostatic fashion via a balance between synthesis and degradation. Many external insults, however, can cause the ER stress which disrupts the homeostasis of proteins in the ER and causes the accumulation of misfolded or aggregated proteins therein. Cells are endowed with three ER stress sensors that monitor the folding status of proteins inside the ER, thereby signaling the message to the nucleus. The unfolded protein response (UPR) was thus coined [14] to denote this cross-talk between the ER and nucleus. There are three ER stress sensors: IRE1, ATF6 and PERK [14]–[18]. Those sensors, through their downstream signaling, restore the folding capacity of ER either by activating the transcription of molecular chaperones and antioxidant proteins or by inhibiting the translation of mRNAs. When cells are facing ER stress, IRE1 begins to dimerize and then undergoes phosphorylation to evoke IRE1's innate endonuclease activity [16]–[18]. Activated IRE1 splices XBP1 mRNA to express the functionally active transcription factor XBP1 that induces the upregulation of several ER chaperones [19], [20]. ATF6 is a transcriptional factor that is proteolytically activated by ER stress and modulates thus the expression of ER chaperones. PERK is a serine/threonine kinase that phosphorylates eIF1a, leading to a global inhibition of protein synthesis in the cell1 [14], [20]. Although molecular and physiological roles of ER stress sensors have been extensively examined, fundamental questions remain. For example, although it is well known that all three sensor proteins are activated when the binding of an ER chaperone BiP to the three sensors is displaced by misfolded proteins, it is still unclear how the three sensors display distinct sensitivities to different forms of ER stress [21], [22]. Further, it is unknown how the three ER stress sensors maintain their conformations and functions when they themselves are under ER stress. A line of recent evidence clearly suggests that the UPR and mitochondrial oxidative stress are tightly linked [23]. It is also known that certain mitochondrial proteins (e.g., Bax) directly associate with IRE1 at the ER to participate in the regulation of cellular survival [24]. Those findings suggest the possibility that the microstructure comprising of the ER and mitochondrial membranes (i.e., the MAM) may serve as an important locus for the pro/anti-apoptotic cross-signaling between the two organelles. Because ER stress sensors and Sig-1R chaperones are related to cellular survival and because Sig-1Rs are enriched at the MAM, we hypothesized that Sig-1R chaperones may promote cellular survival by regulating certain ER stress sensors at the MAM when cells are under oxidative/ER stress. We found here that IRE1, but not PERK or ATF6, resides predominantly at the MAM and that the mitochondria-derived reactive oxygen species (ROS) can preferentially activate IRE1 at the MAM. We also found that Sig-1R chaperones at the MAM can stabilize IRE-1 and enhance the cellular survival by prolonging the activation of the IRE1-XBP1 signaling pathway.

## Materials and Methods

### Cell cultures and treatments

Cells were maintained at 37°C with 5% CO2 under saturated humidity in the following medium: CHO and Neuro 2A (ATTC) in minimum essential medium Alpha medium (MEMAM) with 10%FCS, HeLa and Du145 in Dulbecco's modified Eagle's medium (DMEM) with 10%FCS, and AR42J (ATTC) in DMEM with 20% FCS. The medium was changed every 1–2 days. For drug treatments, cells seeded in 6-well plates or 6–10 cm dishes were incubated with drugs dissolved in distilled H2O or DMSO. Controls received the same amount of the vehicle.

### Western blotting

After treatments with drugs, cells were placed on ice, rapidly washed once with ice-cold phosphate-buffered saline (PBS) and harvested in PBS with a rubber policeman. Cell suspensions were centrifuged at 800×g for 10 min at 4°C. The supernatants were discarded and the pellets were dissolved with 2× Laemmli sample buffer (100 mM Tris-HCl, pH = 6.8, 20% glycerol, 4% SDS) followed by boiling for three min with β-mercaptoethanol and bromophenol blue.

Proteins (15–100 µg) were resolved onto a polyacrylamide SDS-PAGE. Gels were electroblotted onto PVDF membranes (Bio-Rad, Hercules, CA) in Towbeen buffer (25 mM Trisbase, 192 mM glycine) without methanol. Membranes were blocked with 10% non-fat dry milk (Bio-Rad) in Tris-buffered saline plus 0.05% Tween-20 (TBS-T) for 1 h at room temperature. Membranes were incubated overnight at 4°C with an antibody in TBS-T, and probed with the secondary anti-rabbit or anti-mouse IgG conjugated with horseradish peroxidase (HRP) for 1 h at room temperature (1∶1000 dilution). Protein bands were visualized with a SuperSignal West Pico reagent (Pierce Biotechnology) with a Kodak Image Station 440 CF (Kodak, New Haven, CT).

Anti-IRE1, anti-phosphoPERK, ERK (Santa Cruz Biotechnology, CA), anti-FLAG (Sigma, St. Louis, MO), anti-V5, anti-ATP synthase inhibitor (Invitrogen, Carlsbad, CA), antieIF2a (Cell Signaling, Danvers, MA), anti-nucleoporine p63, anti-cytochrome c (BD Transduction Laboratories, Lexington, KY), anti-ERp57, anti-mitofusin-2 (abcam, Cambridge, MA ), and anti-GFP (Clontech, Mountain View, CA) were purchased respectively. The polyclonal anti-Sig-1R antibody was produced by immunizing rabbits with synthetic antigenic peptides [10], [12]. Anti-ATF6 antibody was provided by Dr. K. Mori (Kyoto University, Japan).

For IRE1 immunoprecipitation, cell pellets were lysed in 300 µl of lysis buffer (20 mM HEPES, pH = 7.5, 150 mM NaCl, 1% Triton X-100, 10% glycerol, 1 mM EDTA 10 mM sodium pyrophosphate, 1 mM α-glycerophosphate, 1 mM Na3VO4, 20 mM NaF, 1 mM phenylmethylsulfonyl fluloride, 4 mg/ml aprotinin). Cell lysates were cleared by centrifugation at 15000×g at 4°C for 10 min. Anti-IRE1 antibody (1∶50) was added to the supernatant followed by an incubation overnight at 4°C. Thirty µl of 50% protein A slurry (Amersham, Piscataway, NJ) was added to immune complexes followed by incubation for 90 min at 4°C. Immunoprecipitants were washed twice with lysis buffer followed by boiling for 2 min in 2× sample buffer. In co-immunoprecipitation assays, cells were crosslinked with DSP at 4°C for 30 min prior to cell lysate preparation.

### XBP1 Splicing Assay

Total RNA was prepared by using the NucleoSpin RNA II kit (Macherey-Nagel, Inc., Bethlehem, PA). The reverse transcription (RT)-PCR for XBP1 mRNA was performed by using Titanium one-step RT-PCR kit (Clontech) with 1 µg of total RNA and 50 µl of the reaction mixture under the following thermal cycle: 50°C for 60 min, 94°C for 5 min, 21 cycles at 94°C (30 s), 57°C (30 s), 68°C (60 s), followed by 68°C for 2 min and 4°C (GeneAmp PCR System 9700; Applied Biosystems, Foster City, CA). XBP1 mRNA was amplified by primers 5′-AAACAGAGTAGCAGCGCAGAC-3′ and 5′-GGGATCTCTAAGACTAGAGGC-3′. PCR products were analyzed by 1.5% agarose gel electrophoresis followed by imaging with Image Station 440CF (Kodak IBI, New Haven, CT) under UV light.

### Measurement of fluorescence intensity

After transfection of F-XBP1 ΔDBD-venus to CHO cells, the fluorescence intensity was measured by a fluorescence microplatereader (Perkin-Elmer Victor 3: emission at 525 nm, excitation at 495 nm) in phenol red-free RPMI 1640 medium. Increase of fluorescence intensity induced by ER stress was normalized to the control intensity (no treatment).

### Preparation for the MAM fraction

The MAM fraction was prepared as described elsewhere [6], [10]. Briefly, following homogenization of 2.5×10 cells, nuclear, crude mitochondrial, and microsomal fractions were prepared by differential centrifugation. Supernatants were collected as the cytosolic fraction. The crude mitochondrial fraction in the isolation buffer (250 mM mannitol, 5 mM HEPES, 0.5 mM EGTA, pH 7.4) was subjected to a Percoll gradient centrifugation for separation of the MAM from mitochondria. The activity of phosphatidylserine synthase in each fraction was measured as described previously [10].

### Construction of Expression Vectors

Constructions of Sig-1R siRNA used for CHO cells were described previously [10], [12]. The entire coding region of the human IRE1 or lumenal domain (ΔIRE1) was amplified by PCR with primers with overhang sequences of EcoR1 or Kpn1: 5′-GAATTCATGCCGGCCCGG-3′ and 5′-GGTACCGAGGGCGTCTGGAGT-3′ for IRE1; 5′-GAATTCATGCCGGCCCGG-3′ and 5′-GGTACCATGCATGCTCAGGGG3′ for IRE1. The PCR products were subcloned into the pcDNA3.1 vector (Invitrogen) for expressing V5-tagged IRE1. For expression of FLAG-tagged IRE1, the cDNA fragment was digested from the pcDNA3.1 vector with EcoRI and KpnI, followed by ligation into the pFLAG vector (Sigma). Full length and truncated Sig-1R lacking the ER luminal domain were also subcloned into the pcDNA3.1 vector containing a cDNA encoding a downstream 14 a.a. epitope-tag V5. All epitope tags used were fused to the C-terminus of overexpressed proteins. For transfections, the vector (0.5–4 µg per well)/Lipofectamine-2000 (6 µl per well) (GIBCO) mixture was applied to CHO cells in 6-well plates. After incubation for 5–6 h, the medium was replaced. siRNAs against IRE1, Sig-1R, and mitofusin-2 were purchased from Thermo Scientific (Worcester MA). The pcDNA4-LDLR-G544V vectors provided by Dr. Tveten (Oslo University, Sweden) were transfected into CHO cells stably expressing Tet repressor proteins. The LDL receptor mutants were induced by adding tetracycline to the culture medium (16 hrs, 1 µg/ml).

#### Site-directed Mutagenesis

Site-directed mutagenesis by overlapping PCR was performed to generate single mutants in human IRE1 and/or ΔIRE1 (Clontech). The constructs with mutations at K599A or D123P were thus obtained and used for transfections.

### Immunocytochemistry and Confocal Microscopy

CHO cells were transfected with Lipofectamine-2000 and cultured on poly-D-lysine-coated coverglass in MEM containing 10% FCS. Mito-DsRed, Sig-1R-V5 or IRE1 vectors were used at 0.5 µg/well (12-well plate). Immunocytochemistry was performed as described [10], [12] . UltraView confocal microscopic system (PerkinElmer) was used for imaging.

### Pulse-chase experiment

Sig-1R or control siRNA was transfected 2 days before experiments. Cells seeded on 6-well plates were washed and incubated for 30 min at 37°C in Dulbecco's modified Eagle's medium lacking methionine (Met). The cells were pulsed by adding 200 µCi/ml [ S]Met (Translabel, ICN, Irvine, CA) for 10 min at 37°C. For chasing, the cells were washed three times with MEM followed by incubation in culture medium containing 0.5 mM cold-Met. Chasing was terminated by washing cells with ice-cold PBS. IRE1 in S-Met-labeled CHO cells were immunoprecipitated for autoradiography and immunoblotting.

### Sucrose fractionation for protein aggregates

CHO cells were lysed in lysis buffer. Lysates (1 vol) were layered on the top of sucrose gradients (10%–30%, 5 vol.) and centrifuged at 180,000× g for 17 hr. Twelve fractions were collected from the top for IRE1 immunoprecipitation.

### ΔIRE1-V5/His protein purification and test in vitro for association with Sig-1R

The Ni NTA-agarose resin was first equilibrated with binding buffer (50 mM Tris-HCl, pH 8.0, 500 mM NaCl, 10 mM imidazole) to which the extract of CHO cell expressing ΔIRE1-V5/His was added. The mixture was incubated at 4°C with gentle vertical rotation for 60 min and poured thereafter onto a column. The resin was washed extensively with binding buffer and then with washing buffer (50 mMTris-HCl, pH 6.0, 500 mM NaCl, 10 mM imidazole). Non-specific protein was eluted with 100–300 mM imidazole. The ΔIRE1 protein was eluted with 350 and 500 mM imidazole. A portion of purified ΔIRE1-V5/His was treated with 1 µM β-mercaptoethenol (β-mer) in order to prevent the dimerization. ΔIRE1-V5/His immobilized onto the Ni-column were incubated with purified GST-Sig-1R116–223 [10]. After extensive washings, ΔIRE1 was eluted from the column, and IRE1-Sig-1R complexes were assayed by Western blotting.

### Apoptosis

Tg-induced apoptosis were labeled with annexin V (Invitrogen) or Hoechst 33342. Twenty-four or 16 hrs after administration of Tg (1 µM), the percentage of apoptotic cells were counted in fields when cells were at 100% confluency. At least 50 cells were counted in each well of a 12-well plate where cells were cultured and treated with drugs. Six to seven wells/group were used for assessments.

### Statistical analysis

One-way ANOVA followed by Tukey test or two-way ANOVA followed by Bonferroni's post hoc test was used. A P value of <0.05 was considered to reflect a significant difference. Data were presented as means ± SEM.

## Results

### IRE1 localizes mainly at the MAM

**Figure 1:**
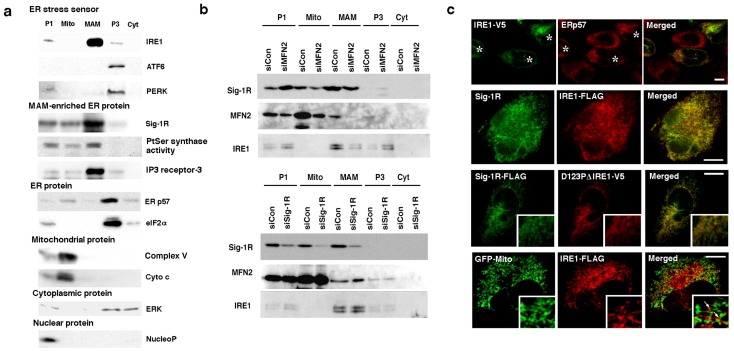
IRE1 localizes at the MAM. (a) The subcellular distribution of ER stress sensors. All endogenous proteins were prepared from wild-type non-stressed CHO cells. P1, nuclear; Mito, mitochondrial; P3, microsomal; Cyt, cytosolic fractions. NucleoP, nucleoporin p62; Complex V, complex V ATP synthase inhibitor; Cyto c, cytochrome c; ERp57, ER thiol-disulfide oxidoreductase p57; ERK, extracellular signal-regulated kinase. Phosphatidylserine (PtSer) synthase activity was measured by the autoradiographic measurement of ^14^C-PtSer as described [10]. All other proteins were measured by immunoblotting. (b) The subcellular distribution of IRE1 in CHO cells with reduced expression of mitofusin-2 (MFN2) or Sig-1Rs. Control (siCon) or specific siRNAs (siMFN2, siSig-1R) were transfected two days before the membrane fractionation. (c) Confocal microscopic observation of the subcellular distribution of Sig-1Rs and IRE1 in CHO cells. In top panels, endogenous ERp57 and transfected full-length IRE1-V5 were immunostained. Asterisks indicate CHO cells transfected with IRE1-V5 (Note: no V5 immunoreactivity in non-transfected cells, verifying the high selectivity of V5 immunostaining). In bottom panels, GFP targeting mitochondria was expressed by gene transfection. Arrows indicate clusters of IRE1-FLAG apposing mitochondria. Scale = 10 µm. Insets on a 5× magnification.

To examine whether ER stress sensors exist at the MAM of ER membranes, we employed the Percoll gradient fractionation in a differential centrifugation. The results indicated that IRE1 exists mainly in the MAM fraction in a fashion similar to that seen with the MAM-enriched ER chaperone Sig-1R (Fig. 1a). In contrast, PERK and ATF6 existed mostly in the P3 microsomal fraction (Fig. 1a). Mitofusin-2 is a molecule that tethers the mitochondrial outermembrane to the ER. The deletion of mitofusin-2 disrupts the association of the MAM with mitochondria [25]. We found that the knockdown of mitofusin-2 in CHO cells (supplemental Fig. S1a) caused a redistribution of IRE1 from the MAM to the P1 nuclear or the P3 microsomal fraction (Fig. 1b). The knockdown of mitofusin-2 per se caused neither apoptosis nor phosphoryaltion of IRE1. In contrast, the subcellular distribution of IRE1 was not significantly changed by the ER stress inducer thapsigargin (Tg, 1 µM, 60 min, data not shown) or the knockdown of Sig-1Rs (Fig. 1b).

The subcellular distribution of IRE1 was also examined by confocal microscopy. Immunocytochemistry under a lower magnification showed that IRE1 and the ER marker ERp57 share a similar cytoplasmic distribution, indicative of their ER localization (Fig. 1c; top panels). At a higher magnification, however, IRE1 (both full-length IRE1 and the IRE1 mutant lacking both the cytoplasmic domain and the dimerization capability, i.e., D123P-ΔIRE1) existed at the ER as punctates that colocalized with the MAM protein Sig-1Rs (Fig. 1c; third level panels). IRE1-enriched punctates in fact directly apposed mitochondria (Fig. 1c; fourth level panels). Specificities of the immunostaining of Sig-1Rs and the FLAG-tagged IRE1 were demonstrated in Supplemental Fig. S1b. Those data indicate that IRE1 predominantly localizes at the MAM of the ER membrane.

### Sig-1Rs regulate the stability of IRE1 and affect thus the level of phosphorylated IRE1 when cells are under ER stress

**Figure 2:**
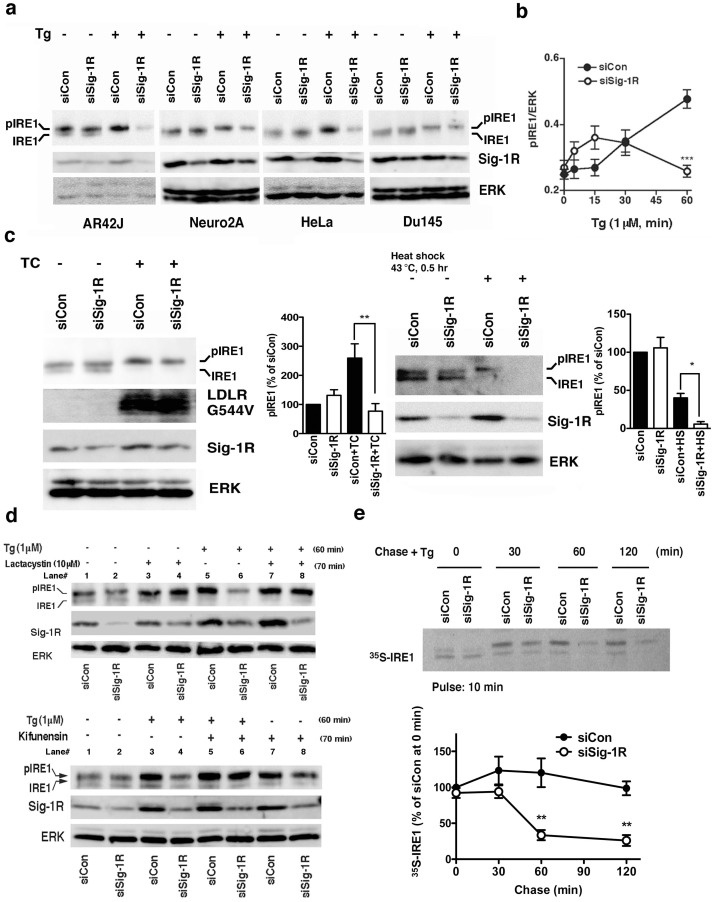
Sigma-1 receptors stabilize IRE1. (a) Sig-1R knockdown decreases phosphorylated IRE1 (pIRE1) in various types of cells when cells are under ER stress. Two days after the transfection of control siRNA (siCon) or Sig-1R siRNA (siSig-1R), cells were treated with thapsigargin (Tg) at 1 µM for 60 min. IRE1 were immunoprecipitated from 60–1000 µg of total protein lysates. (b) The temporal course of pIRE1 levels during ER stress. Control or Sig-1R siRNA was transfected to CHO cells two days before Tg. pIRE1 was measured by immunoprecipitation. The level of pIRE1 (partially phosphorylated IRE1 plus hyperphosphorylated IRE1) was normalized to ERK. The graph represents the means±S.E.M. ***p<0.001 (n = 6). (c) Effect of the Sig-1R knockdown on the level of pIRE1 in CHO cells either overexpressing LDL receptor G544V mutants or being under the heatshock treatment. LDL receptors G544V (LDLR G544V) were induced by the tetracycline treatment for 16 hrs (TC; see Methods). The level of pIRE1 was measured by immunoprecipitation and was normalized to ERK. Graphs represent means±S.E.M (n = 4). *p<0.05, **p<0.01. (d) Effects of lactacystin (10 µM; applied 10 min before Tg) or kifunensin (2 µg/ml; applied 10 min before Tg; lower panels) on Tg (1 µM for 1 hr)-induced decrease of pIRE1 in Sig-1R knockdown CHO cells. (e) Pulse-chase experiment measuring the effect of Sig-1R knockdown on the life-time of IRE1 when cells were under ER stress. After labeling with ^35^S-methionine, CHO cells transfected with control or Sig-1R siRNA were chased in the presence of Tg (1 µM) for indicated periods of time. IRE1 was immunoprecipitated and then detected by direct autoradiography. The graph represents mean±S.E.M (n = 4). **p<0.01 compared with siCon at the same time point.

Since both Sig-1Rs and IRE1 exist at the MAM, we examined whether Sig-1Rs regulate the activation of IRE1 when cells are under ER stress. IRE1 typically displayed two bands in Western blotting in non-stressed CHO or AR42J cells (e.g., lane 1 in Fig. 2a, c). The lower or upper band of IRE1 represents respectively the non-phosphorylated or partially phosphorylated IRE1 (pIRE1) since the alkaline phosphatase treatment decreased the upper band with a concomitant increase of the lower band (Supplemental Fig. S1c). In unstressed HeLa or Du145 cells, the non-phosphorylated IRE1 appeared to be more predominant than the pIRE1 (Fig. 2a). Interestingly, the ER stressor thapsigargin (Tg) caused a slight upward shift of the pIRE1 and concomitantly increased the band intensity (i.e., hyperphosphorylated IRE1) in all types of cells examined here (Fig. 2a). Tg unanimously decreased the non-phosphorylated lower band (Fig. 2a). Notably, in all types of cells tested, the knockdown of Sig-1Rs significantly decreased the level of pIRE1 60 min after the Tg treatment (Fig. 2a, b). Further, we found that, at the very early time point of ER stress (15 min after Tg), the knockdown of Sig-1Rs tended to potentiate the phosphorylation of IRE1 but later in time led to a significant decrease of pIRE1 (Fig. 2b). The decrease of pIRE1 caused by the knockdown of Sig-1Rs at the later time point is unlikely due to the dephosphorylation of pIRE1 because we never saw an increase of dephosphorylated IRE1 during the Tg treatment. The Sig-1R knockdown affected only the IRE-1 activation but not the activation of ATF6 or PERK (Fig. S1d).

The ER stress is also known to be induced by the protein unfolding that arises from certain physiological conditions and not from exogenous stressors like Tg. For example, the class 2a mutant of the LDL receptor that causes familial hypercholesterolemia in humans is physiologically prone to misfolding and can thus lead to the activation of the IRE1 pathway [26]. When the LDL receptor mutant (G544V) was induced by tetracycline in CHO cells (see Method), the receptor mutant caused an increase of pIRE1 (Fig. 2c, lane 1 *vs* lane 3). Similar to that seen in Tg-treated cells, the knockdown of Sig-1Rs also caused a decrease of pIRE1 in cells expressing LDL receptor mutants (Fig. 2c, lane 2 *vs* lane 4). The conventional cellular stressor heatshock was recently shown to activate the IRE1 pathway [27]. Thus, we also employed the heatshock in this study. We found that although heatshock could induce the phosphorylation of IRE1 in CHO cells, the level of pIRE1 was lower than the level that one would expect (Fig. 2c). This result suggested that IRE1 proteins might be unstable when under the heatshock stress. Nevertheless, in heat-treated CHO cells, we saw a further decrease of pIRE1 in Sig-1R knockdown cells (Fig. 2c). Thus, Sig-1Rs indeed could regulate the activation of IRE1 under physiologically or biologically relevant conditions.

Since the Sig-1R is a molecular chaperone at the MAM, we reasoned that the decrease of pIRE1 in Sig-1R knockdown cells may involve the misfolding and subsequent degradation of IRE1. The proteasome inhibitor lactacystin indeed prevented the decline of the pIRE1 in Tg-treated Sig-1R knockdown cells (lanes 6 vs. 8 in upper panels of Fig. 2d). Another proteasome inhibitor MG132 also yielded the same result (Fig. S2a). The inhibitor of ER-associated degradation (ERAD), kifunensine, similarly blocked the decline of pIRE1 under the Tg treatment condition (lanes 4 vs. 6 in lower panels of Fig. 2d). Those results suggest that without Sig-1Rs IRE1 is vulnerable to proteasomal degradation that might involve the ERAD. The sucrose fractionation on the Triton X-100 cellular lysates also demonstrated that Tg caused IRE1 to exist in high-density fractions (i.e., tendency to aggregate) in Sig-1R knockdown cells but not in control cells (Fig. S2b).

Results from the pulse-chase experiment indicated that the knockdown of Sig-1Rs did not affect the half-life of IRE1 in non-stressed cells (Supplemental Fig. S2c). In contrast, when cells were under ER stress, the knockdown of Sig-1Rs significantly shortened the half-life of IRE1 (Fig. 2e). Interestingly, IRE1 at the MAM has a longer half-life than IRE1 at the non-MAM area (i.e., microsome) where there are less Sig-1Rs (Supplemental Fig. S2d). Taken together, those results suggest that Sig-1Rs at the MAM stabilize IRE1 and prevent IRE1 from proteasomal degradation only when cells are under ER stress. Those findings also suggest that IRE1, when under ER stress, might be chaperoned by Sig-1Rs at the MAM.

### Temporal pattern of the IRE1-Sig-1R association

**Figure 3:**
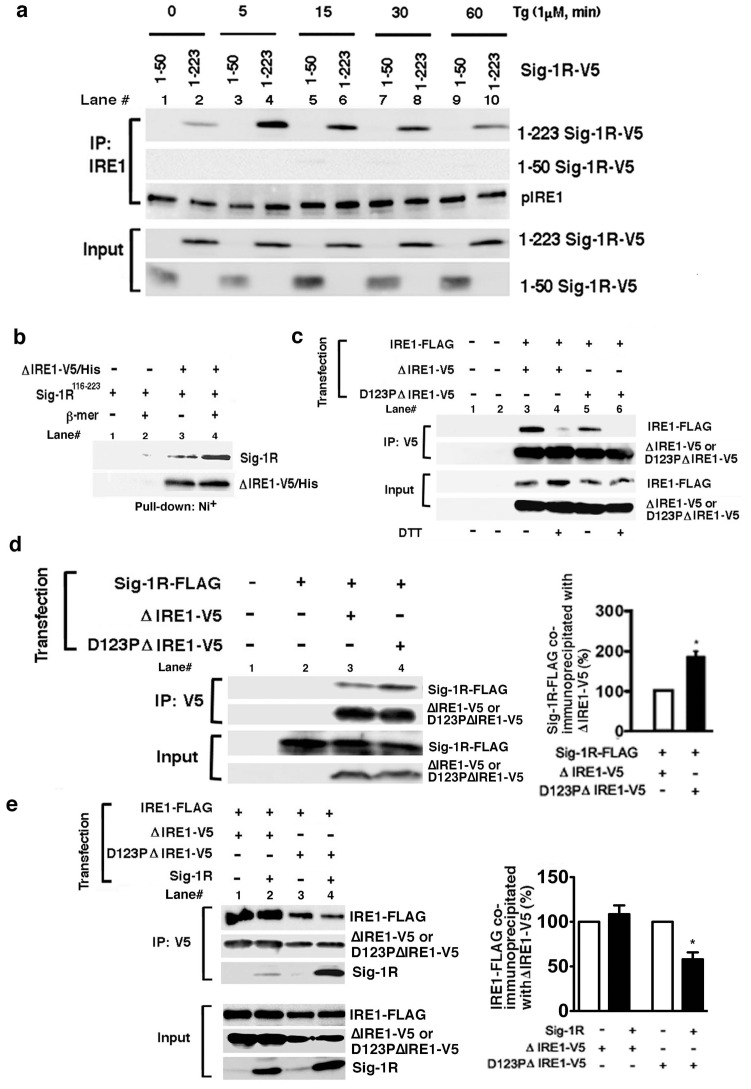
Sigma-1receptors preferentially associate with the monomeric form of IRE1 in the lumen of the ER. (a) Time-dependent association of V5-tagged Sig-1Rs with IRE1 during ER stress. IRE1 was immunoprecipitated in CHO cells expressing V5-tagged full-length Sig1R-V5 (1–223) or truncated Sig-1R-V5 (1–50). Thapsigargin (Tg) was applied to the medium for indicated periods of time. (b) Direct association between purified ΔIRE1-V5/His and the ER lumenal domain (116–223) of Sig-1Rs (GST-Sig-1R116–223) in vitro. Purified ΔIRE1 immobilized on a Ni^+^-column was incubated with purified GST-Sig-1R116–223 polypeptides. After extensive washing, the GST-Sig-1R116–223 associating with ΔIRE1-V5/His was measured by Western blotting. β-mer (+): the purified ΔIRE1-V5/His was pretreated with β-mercaptoethanol to prevent the dimerization of ΔIRE1-V5/His. The image represents three independent experiments. (c) Involvement of both the ionic bond (D123 site) and the disulfide bonds in the dimerization of IRE1. To assess the dimerization of IRE1, FLAG-tagged full-length IRE1 was co-immunoprecipitated with ΔIRE1-V5 with or without a point-mutation at D123 (e.g., ΔIRE1, D123PΔIRE1). Dimerization of IRE1, via disulfide bonds, was disrupted by DTT (20 mM for 30 min). (d) Association of Sig-1Rs with IRE1 depends on the dimerization status of IRE1. V5-tagged ΔIRE1 with or without a point-mutation at D123 were immunoprecipitated. The association of Sig-1R with Δ IRE1-V5 was assessed by measuring co-immunoprecipitated Sig-1R-FLAG. The graph represents means±S.E.M. *p<0.05 (n = 4). (e) Effects of overexpressed Sig-1Rs on the dimerization of IRE1. Dimerization of IRE1 was assessed by measuring IRE1-FLAG co-immunoprecipitated with V5-tagged IRE1 (ΔIRE1-V5 or D123PΔIRE1-V5). Note: overexpressed Sig-1Rs co-immunoprecipitated significantly more with IRE1-V5 when IRE1 was mutated at D123. The graph represents means±S.E.M (n = 4, *p<0.05).

To clarify whether IRE1 serves as a substrate of the Sig-1R chaperone, the physical interaction between the two molecules were examined. Immunoprecipitation studies showed that IRE1 and full-length Sig-1R (1–223) associated at minimal level under the basal condition but the association was largely intensified when cells were under ER stress (Fig. 3a). The association of Sig-1Rs with IRE1 apparently reached the maximum in 5 min after the Tg treatment. Majority of IRE1 at the 5 min time point in fact were not yet hyperphosphorylated (see Fig. 2b, the closed circle) or, in other words, were not yet dimerized for further phosphorylation. Those results suggested that the maximum association between IRE-1 and Sig-1Rs took place when the IRE1 remains in monomeric form. Further rendering support to this notion was the fact that the co-immunoprecipitation between IRE-1 and Sig-1Rs decreased at the 60 min time point after the Tg treatment (Fig. 3a) when IRE1s were completely dimerized and phosphorylated (Fig. 2b). We also demonstrated that endogenous Sig-1Rs and endogenous IRE1co-immunoprecipitated at the 5 min time point after the Tg treatment (Fig. S3a). However, Sig-1Rs without the ER lumenal domain (i.e., Sig-1R 1–50a.a.) could not associate with IRE1 (Fig. 3a). The direct interaction between Sig-1Rs and IRE1 was also tested by using purified proteins in vitro. Purified ER luminal domain of IRE1 with a V5/His tag (ΔIRE1-V5/His; note: majority of purified ΔIRE1-V5/His form dimers, see Supplemental Fig. S3b) pulled down a marginal amount of the purified GST-tagged ER lumenal domain of Sig-1Rs (Sig-1R116–223; lane 3 in Fig. 3b). However, when the purified ΔIRE1-V5/His remained as monomers by a pretreatment with β-mercaptoethanol more of the Sig-1R116–223 were pulled down by ΔIRE1-V5/His (Fig. 3b). Those data thus suggest that Sig-1Rs directly and preferentially associate with the monomeric form of IRE1 via the ER lumenal domain.

### The Sig-1R uses its chaperone segment in the ER lumen to interact with IRE1 monomers and dictates thus the temporal course of IRE1 activation: IRE1 mutant study

To further confirm whether Sig-1Rs may preferentially bind the IRE1 monomer, we performed co-immunoprecipitation studies by using IRE1 mutants that are slower or difficult to form dimers when compared to the wild-type IRE1. The ER lumenal domain of IRE1 contains two types of bonds that are essential for dimerization: the disulfide bond between Cys 148 and Cys 332 [28], and the ionic bond at D123 [29]. As expected, when either of those IRE1 bonds was disrupted respectively by dithiothreitol (DTT) or by the point-mutation at D123, the full-length wild-type IRE1-FLAG (IRE1-FLAG) that could co-immunoprecipitate with the IRE1 mutants (ΔIRE1-V5) decreased significantly (Fig. 3c top panels, lanes 3 vs. 4 for DTT, lanes 3 vs. 5 for the D123P mutation). When DTT and the point mutation were combined, the two IREs failed to associate (lane 6 in Fig. 3c). Thus, the dimerization of IRE1 relies solely on those two types of bonds. Next, we compared the binding capability of Sig-1Rs to ΔIRE1 that is with or without the D123 mutation. The mutation at this motif of the ΔIRE1 apparently increased the mutant's association with Sig-1Rs (Fig. 3d top panel, lane 3 vs. 4; See also the accompanied graph), and this association (D123P ΔIRE1-Sigma-FLAG) was potently suppressed by overexpression of Sig-1Rs (Supplemental Fig. S4a). In agreement with this result, the pulse-chase experiment also showed that the D123P ΔIRE1, which might possess a higher affinity for Sig-1Rs, has a longer life-time than that of ΔIRE1 (Supplemental Fig. S4b). Similarly, when the dimerization of IRE1 was disrupted by the DTT treatment, the association of Sig-1Rs with ΔIRE1 increased (Fig. S4c). The point mutation at the kinase domain of IRE1 (K599), however, did not affect the association (Fig. S4d). Therefore, the monomeric state of IRE1 determines the IRE1-Sig-1R association.

Because Sig-1Rs associate with IRE1 as early as 5 min after the Tg treatment (Fig. 3a) and because the chaperone Sig-1R preferentially binds IRE monomers (Fig. 3b), we speculated that the association of Sig-1Rs with the monomeric IRE1 may interfere with and thus transiently inhibit the dimerization of IRE1. We therefore examined next if the Sig-1R binding to IRE1 may reduce the dimerization of IRE1. To keep the test system simple, we decided to use cells without imposing ER stress on them. Further, in order to track the IRE1 dimerization, we overexpressed IRE1 with two different tags: FLAG or V5. Overexpressed IRE1 *per se* is known to undergo autodimerization in non-stressed cells [30]. Thus, overexpressing the dimerization-resistant IRE1 analogs (ie., D123PΔIRE1) in non-stressed cells represent some “open” opportunities for Sig-1Rs to bind IRE1. In addition, we also chose to overexpress Sig-1Rs in the system in order to monitor a “semi” dose-dependent effect caused by Sig-1Rs. Results showed that the dimerization between IRE1-FLAG and ΔIRE1-V5 under non-stressed condition was not affected by Sig-1Rs (Fig. 3e top panel, lanes 1 vs. 2). In contrast, the dimerization between IRE1-FLAG and D123PΔIRE1-V5 was reduced by overexpression of Sig-1Rs (lanes 3 vs. 4 in Fig. 3e). Thus, the binding of Sig-1Rs to the IRE1 monomer may transiently inhibit and delay thus the dimerization of IRE1.

### Sig-1R knockdown causes apoptosis by compromising the IRE1-XBP1 signaling

**Figure 4:**
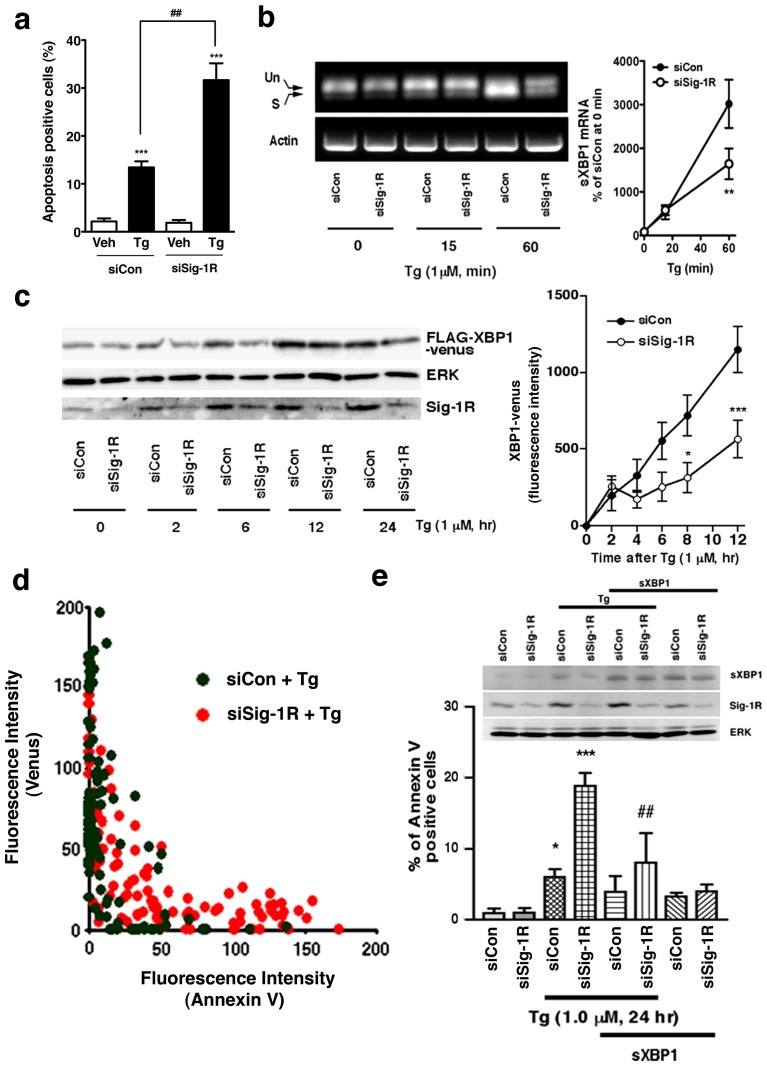
Effect of Sig-1R knockdown on the Tg-stimulated IRE1/XBP1 signaling pathway in CHO cells. (a) Effect of Sig-1R knockdown on Tg-induced apoptosis. Induction of apoptosis by Tg (1 µM, for 24 hr) was quantified by using Hoechst 33342 staining. The percentage of apoptotic cells was measured from 6 individual samples (more than 200 cells were counted in each sample) and is reported as means±S.E.M. ***P<0.001 compared with vehicle (Veh), ##P<0.01 compared with siCont with Tg. (b) Effects of Sig-1R knockdown on the splicing of XBP1 mRNA. CHO cells transfected with control or Sig-1R siRNA were treated with Tg for indicated periods of time. Total RNA was extracted and XBP-1 or actin transcripts were amplified by RT-PCR. Un, unspliced; S, spliced; sXBP1; spliced XBP1. The graph represents means±S.E.M. **p<0.01 compared with siCon at the same time point (n = 4). (c) Effects of Sig-1R siRNA on the expression of FLAG-XBP1-venus fusion proteins. CHO cells transfected with FLAG-XBP1-venus plasmids were treated with Tg, and the expression of FLAG-XBP1-venus was measured by Western blotting (left panels) or a fluorescence microplate reader (the right graph). *p<0.05, ***p<0.001 compared with siCon at the same time point (n = 8). Note that venus is expressed only when FLAG-XBP1 mRNA is spliced by active IRE1. (d) Sig-1R siRNA enhanced apoptosis in Tg-treated cells negatively correlates with the activity of XBP1 mRNA splicing. Fluorescence intensities (annexin V vs. venus) of individual CHO cells were plotted in the graph. Control siRNA (siCon) in green, Sig-1R siRNA (siSig-1R) in orange. (e) Overexpression of spliced XBP1 attenuates apoptosis enhanced by Sig-1R knockdown in Tg-treated cells. Each bar represents the means±S.E.M. (n = 6–7 samples; >50 cells/sample were counted). *P<0.05, ***P<0.001 compared with siCon alone. ##P<0.01 compared with siSig-1R with Tg.

In agreement with our previous report [10], we confirmed that the knockdown of Sig-1Rs potentiates the apoptosis when cells are under ER stress (Fig. 4a). Those results indicate a cellular protective action of Sig-1Rs. Inasmuch as Sig-1Rs affect the activation of IRE1, we examined here if Sig-1Rs may promote cellular survival via the IRE1-XBP1 signaling pathway. We found that in Sig-1R knockdown cells the splicing of XBP1 mRNA, especially at 60 min after the Tg treatment, was significantly reduced (Fig. 4b). We further confirmed this effect of the Sig-1R knockdown on the level of XBP1 by employing the F-XBP1ΔDBD-venus expression system. In this system, XBP1 proteins are fused to fluorescent protein venus only when the XBP1 mRNA is spliced by the activated endogenous IRE1, allowing thus a sensitive and quantitative fluorescence detection of the XBP1 expression [31]. Results showed that the knockdown of Sig-1Rs significantly decreased the XBP1-venus induced by Tg (Fig. 4c). We next examined the relation between the expression level of the XBP1-venus protein and the degree of cellular apoptosis (i.e., annexin V-positive cells). In general, cells expressing higher levels of XBP1-venus often showed a lower degree of apoptosis (Fig. 4d). Sig-1R knockdown cells expressed a lower level of XBP1-venus while exhibited a higher degree of apoptosis (Fig. 4d). Thus, an apparent negative correlation exists between the IRE1 activity and the Tg-induced apoptosis in both control and Sig-1R knockdown cells.

We tested if the overexpression of spliced XBP1 protein may reverse the apoptosis exacerbated by the Sig-1R knockdown. The transient expression of a low level of spliced XBP1 (sXBP1/pcDNA3.1) has been previously shown to benefit the survival of CHO cells [32]. Thus, using a low expression level of spliced XBP1 proteins, we found that the overexpressed spliced XBP1 significantly blocked the Tg-induced apoptosis in Sig-1R knockdown cells (Fig. 4e). Those results, when taken together, confirm that Sig-1Rs at the MAM play an important role in cellular survival via the enhancement of the IRE1-XBP1 signaling pathway.

### Mitochondria-derived oxidative stress is transmitted to the nucleus via the Sig-1R-IRE1-XBP1 signaling axis

**Figure 5:**
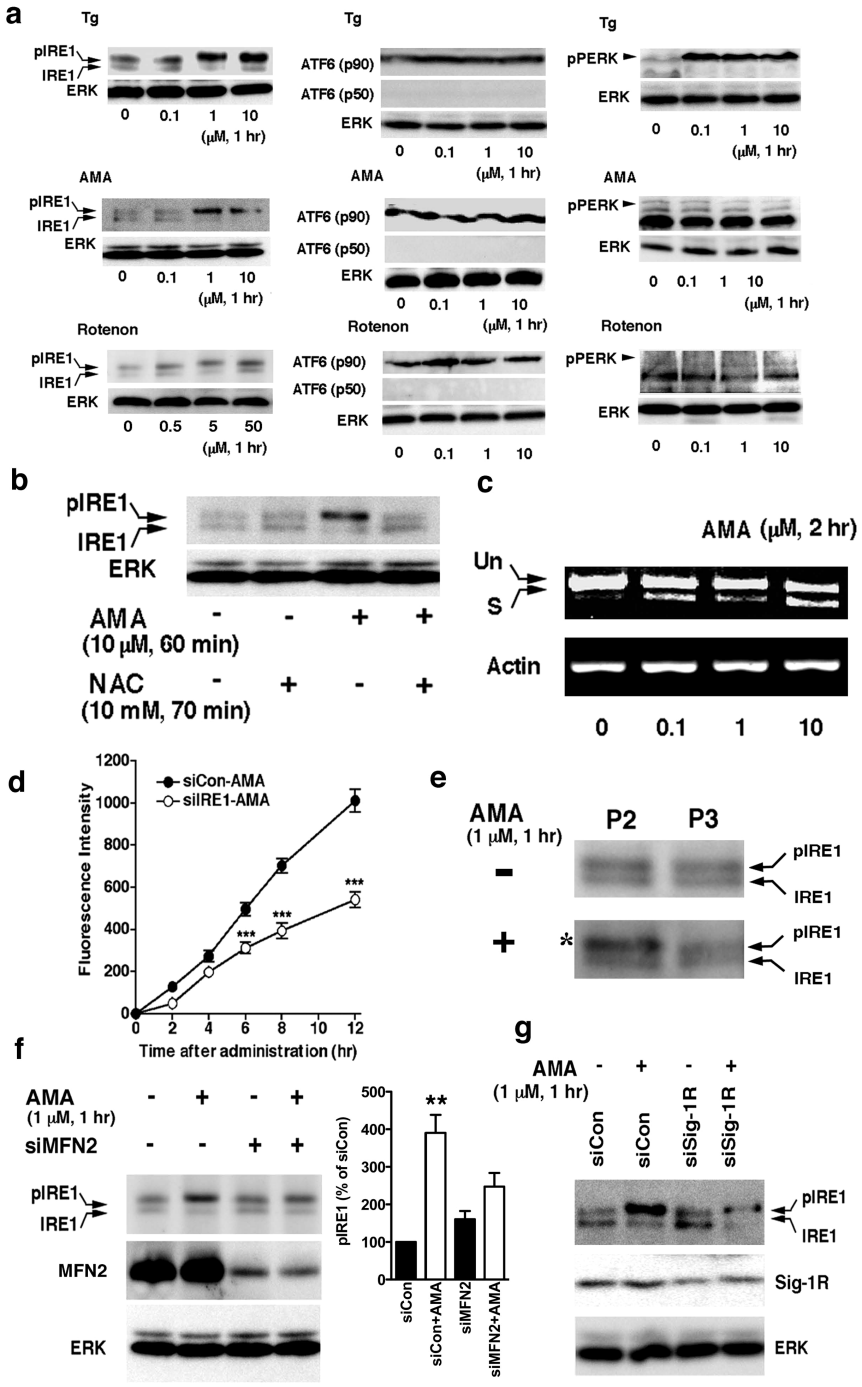
Mitochondria-derived oxidative factor activates IRE1. (a) Effects of Tg, antimycin A (AMA), or rotenone on the activation of ER stress sensors. IRE1 were immunoprecipitated before detection by western blottings. No active form of ATF6 (p50) or phosphorylated PERK was detected under the experimental condition with AMA or rotenone. (b) Effects of NAC on phosphorylation of IRE1 caused by AMA. (c) Effects of AMA on the splicing of the XBP1 mRNA in CHO cells. Un, unspliced; S, spliced. (d) AMA (1 µM) induced the expression of XBP1-venus in an IRE1-dependent manner. CHO cells were transfected with FLAG-XBP1-venus plasmids and siRNA (siCon, scrambled siRNA; siIRE1, IRE1 siRNA) two days before the AMA treatment. Induction of FLAG-XBP1-venus proteins was measured by a fluorescence microplate reader. ***P<0.001 (n = 12), compared with siCon. (e) Selective activation of IRE1 by AMA in the MAM-containing crude mitochondrial fraction. AMA-treated CHO cells were homogenized and subjected to differential centrifugation. The asterisk indicates the hyperphosphorylated form of IRE1. (f) Effect of mifotusin-2 knockdown on the AMA-induced IRE1 phosphorylation. siRNA against mitofusin-2 (siMFN2) or scrambled control siRNA (siCon) was transfected into CHO cells two days before the treatment with AMA. In the graph, IRE1 was normalized to ERK and shown as means±S.E.M. **p<0.01 (n = 4) compared with siCon without AMA. (g) AMA induces a reduction of pIRE1 proteins in CHO cells with reduced expression of Sig-1Rs. Control or Sig-1R siRNA were transfected into CHO cells two days before AMA. IRE1 was immunoprecipitated before detection.

We found in the next series of experiments that in contrast to PERK and ATF6, IRE1 is particularly sensitive to mitochondrion-derived ROS. Antimycin A (AMA; 0.1–10 µM), which generates ROS by inhibiting the complex III of the mitochondrial electron transport chain [33], could cause the phosphorylation of IRE1 (Fig. 5a, left middle panels). Similarly, rotenone, another mitochondrial ROS inducer, activated IRE1 (Fig. 5a, left lower panels). In contrast, AMA or rotenone barely activated ATF6 or PERK. We could not detect the activated form of either ATF6 (as p50) or PERK (as phosphorylated PERK) after the AMA or rotenone treatment (Fig. 5a, middle and right center panels). Tg at 0.1 µM caused the activation of PERK (Fig. 5a, upper right panels) but not IRE1 or ATF6 (Fig. 5a). Thus, different ER stresses apparently activate different ER stress sensors. Because AMA is a specific inducer of mitochondrial ROS and because IRE1 resides mostly at the MAM, those results suggest that IRE1 is preferentially activated by mitochondria-derived ROS at the MAM. In agreement with this notion, we found that the free radical scavenger N-acetyl cysteine (NAC) blocked the phosphorylation of IRE1 caused by AMA (Fig. 5b).

We next tested if the phosphorylation of IRE1 by AMA results in the downstream splicing of XBP1 mRNA that involves perhaps Sig-1Rs. AMA (0.1–10 µM) indeed caused the splicing of XBP1 mRNA (Fig. 5c). This result was also confirmed in vivo by the F-XBP1ΔDBD-venus expression system (Fig. 5d). The effect of AMA promoting the expression of XBP1-venus proteins undoubtedly involves the activation of IRE1 since the action was attenuated by the knockdown of IRE (Fig. 5d). AMA likely activates IRE1 that resides at the MAM because AMA promoted the phosphorylation of IRE1 that was fractionated into the crude mitochondrial fraction (MAM plus mitochondria) but not the IRE1 that was in the microsomal fraction (Fig. 5e). Further, the knockdown of mitofusin-2 that disrupts the ER-mitochondria association also reduced the AMA-induced phosphorylation of IRE1 (Fig. 5f). Lastly, we tested whether the knockdown of Sig-1Rs affects the level of pIRE1 when cells are facing the ROS insult derived from mitochondria. Indeed, in Sig-1R knockdown cells, the level of pIRE1 was significantly reduced by the knockdown of Sig-1Rs when cells were treated with AMA (Fig. 5g). Taken together, those results indicate that the mitochondria-generated ROS selectively activates IRE1 in a Sig-1R-dependent manner and promotes therefore the splicing of XBP1 mRNA. Further, because mitochondria can directly appose the MAM of the ER, those results suggest the existence of a mitochondria-ER-nucleus pathway for the activation of the IRE1 signaling that quenches in part the insult of mitochondrion-derived ROS to the cell.

## Discussion

We found that the mitochondria-derived ROS can activate the IRE1-XBP1 signaling pathway but not the other two ER stress sensors ATF6 and PERK. When cells are under stress or are facing pathological insults, most of the ROS are generated from mitochondria via the oxygen consumption processes [34] although the ER is also known to generate ROS via the oxidative formation of disulfide bonds [35], [36]. Therefore, the MAM of the ER might be exposed to high levels of ROS. From this point of view, it is reasonable to suggest that the IRE1 is strategically placed by nature in the cell at the MAM in order to “sense” in an efficacious manner the insult of mitochondria-derived ROS. A remaining question is of course why the mitochondrion-derived ROS cannot activate ATF6 or PERK which are outside of the MAM. At present, we can only speculate that perhaps because the MAM is well known to be biochemically unique compared to other part of the ER membrane the MAM may have a as yet un-clarified capacity to sequester the mitochondrion-derived ROS. Further investigation is certainly warranted in this regard. In addition, we have previously shown that upon ER stress [10] the Sig-1R dissociates from BiP which also is known to dissociate from IRE1, ATF6, and PERK upon the same stress. The dissociation of Sig-1R from BiP in this regard would unleash the chaperone activity of Sig-1R to chaperone the unfolded IRE1. Taken together with the seemingly selective activation of only IRE1 by the mitochondrion-derived ROS as shown above, those results suggest that the mitochondrion-derived ROS can only cause the dissociation of BiP from the IRE1 and not from ATF6 or PERK. Of course, future experiments will be planned to examine the possibility mentioned as such. Our data nevertheless suggest that the spatial differentiation of the three ER stress sensors might partly explain why they respond differently to different stressors.

**Figure 6:**
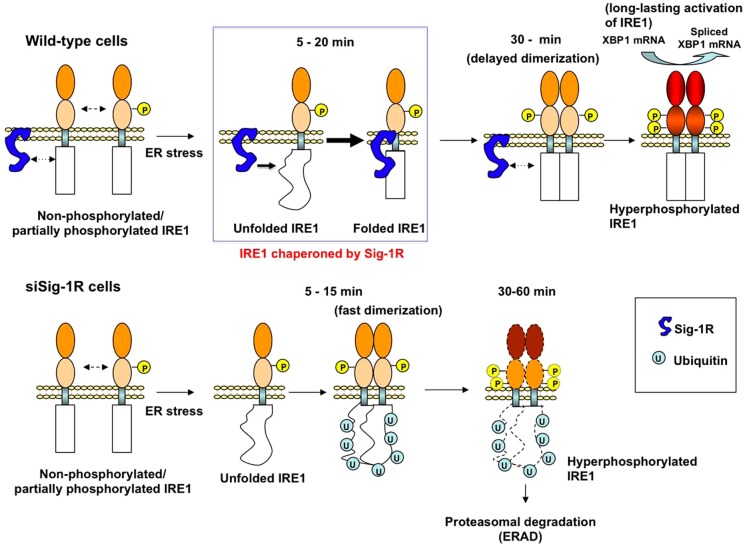
Schematic model depicting the role of the Sig-1R chaperone in the activation of IRE1. The Sig-1R molecular chaperone enhances its association with IRE1 to correct or stabilize the conformation of IRE1 when cells are facing ER stress (i.e., as indicated in the blue-lined rectangle). This transient association of the Sig-1R with IRE1 interferes with the dimerization of IRE1, leading to a delay in the transautophosphorylation of IRE1 (30 min). This delayed dimerization/phosphorylation, however, ensures a long-lasting active form of IRE1 (the cytoplasmic domain filled in red) which splices the XBP1 mRNA. In lower panels, when Sig-1R knockdown cells encounter ER stress, IRE1, although being misfolded, can still quickly dimerize and transautophosphorylate (5–15 min lower panels). The conformationally awry pIRE1, which may still possess endonuclease activity albeit being less compared to controls, is however readily ubiquitinated and degraded by proteasomes.

Heat shock protein Hsp90 was reported to constitutively modulate the stability of IRE1 when cells are not under stressful conditions [37]. However, it has not been reported if a mechanism might exist that provides the stabilization for IRE1 when cells are under stress. We provide here the first report showing that when ER stress causes a conformational change of IRE1 at the MAM, the ER Sig-1R chaperone at the MAM is there to help (see Fig. 6). Although Sig-1R chaperones associate with IRE1 only transiently in the beginning of the ER stress (e.g., 5–15 min after Tg), the transient association apparently is sufficient to establish a conformationally stable and long-lasting IRE1 endonuclease (Fig. 2b, Fig. 6). The transient association of Sig-1Rs with IRE1 may cause a delay for the subsequent dimerization/phosphorylation of IRE1 (Fig. 6). The delay may represent an in vivo process during which the Sig-1R chaperone acts on its client, here being the stressed IRE1.

Compared to that seen in wild type cells, the IRE1 in Sig-1R knockdown cells is largely degraded at the 60 min time point after Tg (Fig. 2e). As a result, the low level of IRE1 in Sig-1R knockdown cells at the 60 min time point after Tg corresponded strikingly to the low level of the resultant XBP-1 mRNA splicing (Fig. 4b). It is noteworthy that, on the contrary, at the 15 min time point after Tg treatment, the IRE1 protein levels are the same between the wild type and Sig-1R knockdown cells (Fig. 2e), suggesting that the IRE1 in Sig-1R knockdown cells was not yet degraded by proteasomes. At the same 15 min time point, however, although the IRE1 was highly phosphorylated in Sig-1R knockdown cells (Fig. 2b) there was no apparent increase in the XBP-1 mRNA splicing (Fig. 4b). This apparent dilemma can be potentially explained as follows: (1) At the 15 min time point in wild type cells, the IRE1 is not yet dimerize to become active (because the Sig-1R chaperone is doing its work on the IRE1 monomers), and, as a result, not much XBP-1 mRNA splicing activity could take place; (2) On the other hand, at the 15 min time point in Sig-1R knockdown cells when the IRE1 protein was not yet degraded by proteasomes (Fig. 2e), IRE1 could dimerize, albeit being conformationally awry, and may possess some endonuclease activity to splice the XBP-1 mRNA (Fig. 4b).

Protein misfolding in the ER can cause UPR, which plays important roles in the etiology of numerous diseases [20], [22] including neurodegenerative diseases [38]. Specifically, previous reports have suggested that neuronal death in Alzheimer's disease, Parkinson's disease, or ischemia involves dysfunction of the ER [39]–[42]. ER stress sensors play a double-edged role in cell survival or cell death [22]. When the level of unfolded proteins exceeds a threshold, the cell might commit suicide by activating the cell death pathways including those involving the PERK/ATF4 and ATF6/CHOP [18], [20], [22]. In contrast, sustained activation of the IRE1-XBP1 pathway constitutively promotes survival against ER stress [22]. Thus, the sustenance of the IRE1 stability may represent a reasonable means to promote cellular survival. In this regard, it is interesting to note that many preclinical studies have reported the therapeutic efficacies of Sig-1R ligands in ameliorating stroke-or β-amyloid-induced neurodegeneration [8], [43] and in promoting neuroplasticity related to drug abuse [8], . In contrast, mutations on the Sig-1R gene are recently reported to cause amyotrophic lateral sclerosis in juveniles [45]. How exactly Sig-1R ligands might exert the neuroprotective effect is not totally clarified. Given the results shown in the present study, it is not unreasonable to suggest that the Sig-1R chaperones sustaining and prolonging the IRE1-XBP1 signaling pathway may explain the therapeutic efficacy of some Sig-1R ligands in neurodegenerative disorders. Interestingly, reports showed that Sig-1R agonists were effective against stroke even when the agonists were applied several hrs after a stroke episode [46], [47].

In conclusion, our results provide a new mechanism whereby the MAM regulates cellular survival by transmitting the message of ROS from mitochondria to the nucleus as well as by attenuating ER stress via the enhancement of the IRE1 stability by Sig-1R chaperones at the MAM. Our results also render support to an important role of the Sig1R as an interorganelle signaling modulator in the living system [13].

## Supporting Information

Figure S1 **Sig-1R, mitofusin-2, and IRE1, ATF6 or PERK in CHO cells.** (a) Knockdown of mitofusin-2 (MFN2) in CHO cells by siRNA. Scrambled (siCon) or MFN2 siRNA were trabsfected to CHO cells for 2 days. Protein levels were measured by immunoblotting. (b) Specific immunostaining of Sig-1Rs and IRE-1-FLAG in CHO cells. In top panels CHO cells transfected with siSig-1R accompanied by EYFP (asterisks) were immunostained with Sig-1R antibodies. Note that Sig-1R immunoreactivity was seen only in cells without transfection (no EYFP) confirming the specificity of Sig-1R staining. In bottom panels, transfected IRE1-FLAG (asterisks) and endogenous ERp57 were immunostained. Note: no FLAG immunoreactivity in non-transfected cells. (c) Effect of calf intestine alkaline phosphatase treatment (CIAP, 1 hr) on the phosphorylation status of IRE1. IRE1 in CHO cells were immunoprecipitated from cell lysates, and then tratened with CIAP for 1 hr before Western blotting. Note: CIAP decreases the upper band of IRE1 while concomitantly increases the lower band. (d) Effect of Sig-1R knockdown on the activation of ATF6 and PERK in CHO cells. DTT or thapsigargin was applied to activate those ER stress sensors. Activation of PERK was measured by pPERK immunostaining. Intensities of immunoblotted bands were measured and normalized to that of ERK.
(TIF)

Figure S2 **Effects of Sig-1R knockdown on the stability of IRE1.** (a)Effects of MG132 (10 µM; applied 10 min before thapsigargin (Tg) on Tg (1 µM for 1 hr)-induced degradation of IRE1 in Sig-1R knockdown CHO cells. Note that the degradation of IRE1 caused by Sig-1R knockdown in Tg-treated cells (lane 4) was inhibited by MG132 (lane 8). (b) Sig-1R knockdown potentiates the aggregation of IRE1 induced by thapsigargin (Tg). siRNA-transfected CHO cells were stimulated with 1 µM of Tg for 1 hr in the presence of 10 µM of lactacystin (for 70 min). The total cell lysate was prepared with 1% Triton X-100 and subjected to sucrose gradient centrifugation. Twelve fractions were obtained from the top. Note the increase of IRE1 in high-density fractions 9–11 in Sig-1R knockdown cells treated with Tg. (c) Effect of Sig-1R knockdown on the stability of newly synthesized IRE1 in un-stressed CHO cells. CHO cells were pulse-labeled with ^35^S-methionine (^S35^-Met) for 10 min followed by chasing with excess cold methionine in the culture medium. IRE1 in ^S35^-Met-labeled CHO cells were immunoprecipitated followed by autoradiography. Intensities of unphosphorylated IRE1 (left graph) and phosphorylated IRE1 (right graph) were densitometrically measured. Each point represents the means ±s.e.m. (n = 4). (d) Kinetics of IRE1 degradation at P3 (upper panel; the non-MAM ER microsomes) and P2 (bottom panel; crude mitochondrial fraction containing both MAM and mitochondrial fractions. After inhibition of protein synthesis by cycloheximide (5 mM, for 1 hr), Tg (1 µM) or vehicle (“−” sign in the panel) was applied to wild-type CHO cells. After the subcellular fractionation, levels of IRE1 were measured by immunoprecipitation of 20 µg of P2 proteins or 200 µg of P3 proteins. The graph represents the average of three independent experiments with s.e.m. Note that IRE1 at P3 fractions show a faster degradation when compared to the IRE1 at P2 fractions in both of the Tg(−) and Tg(+) samples.
(TIF)

Figure S3 **Association of Sig-1R with full-length IRE1 and the dimerization of IRE1.** (a) ER stress-induced association of Sig-1Rs with IRE1. Before and after the treatment with thapsigargin (Tg), MAM fractions were prepared from CHO cells. Endogenous IRE1 or Sig-1R were immunoprecipitated. Co-immunoprecipitated counter proteins were measured by immunoblotting. Input: immunoblotting of total lysates. (b) Purified ΔIRE1-V5/His constitutively form dimmers under the non-reducing condition. ΔIRE1-V5/His was purified by a Ni+ column chromatography (see Methods). Lanes “−” represent the eluant from a Ni+ column pre-incubated with wild-type lysates (i.e., no expression of ΔIRE1-V5/His); lanes “+” represent the eluant from a column incubated with cell lysates containing overexpressed ΔIRE1-V5/His. Note that most of purified ΔIRE1-V5/His show the dimeric form in the non-reducing condition [i.e., without β-mercaptopethanol (β-mer)].
(TIF)

Figure S4 **Domain interactions between Sig-1R and IRE1.** (a) Evidence for the direct protein-protein interaction between Sig-1R and IRE1 via mass-action law by substituting tagged Sig-1R with overexpression of non-tagged Sig-1R. Effects of overexpressed non-tagged Sig-1Rs on the interaction between tagged Sig-1Rs and IRE1s. D123PΔIRE1 was co-immunoprecipitated with FLAG-tagged Sig-1Rs with or without overexpression of non-tagged Sig-1Rs. (b) The life-time of D123PΔIRE1 and ΔIRE1-V5. CHO cells were pulse-labeled with S35-methionine (35S-Met) for 10 min followed by chasing with excess cold methionine in the culture medium. The ΔIRE1-V5 in S35-Met-labeled CHO cells was immunoprecipitated with anti-V5 antibodies followed by detection by autoradiography. In the graph, band intensities of D123PΔIRE1-V5 at respective chase time were taken as 100% (means±s.e.m.; *p<0.05 by paired t-test, n = 3). (c) Association of Sig-1Rs with IRE1 does not depend on the phosphorylation status of IRE1. FLAG-tagged Sig-1Rs were immunoprecipitated. Co-immunoprecipitated V5-tagged IRE1 or mutant IRE1 (K588A-IRE1-V5) lacking the kinase activity was measured by immunoblotting. (d) Monomers of IRE1 induced by dithiothreitol (DTT) preferentially associate with Sig-1Rs. FLAG-tagged Sig-1Rs and V5-tagged ΔIRE1 (with/without mutation at D123) were expressed in CHO cells for co-immunoprecipitation. Note the increase of Sig-1R-FLAG co-immunoprecipitated with ΔIRE1-V5 by DTT treatment and/or D123 mutation. The DTT treatment did not affect the molecular weight or the level of Sig-1R-FLAG.
(TIF)
