# Supplementary figures and images for: Sigma-1 Receptor Chaperone at the ER-Mitochondrion Interface Mediates the Mitochondrion-ER-Nucleus Signaling for Cellular Survival
Source: PLoS One. 2013 Oct 18;8(10):e76941. doi: 10.1371/journal.pone.0076941 (PMC3799859; doi:10.1371/journal.pone.0076941)

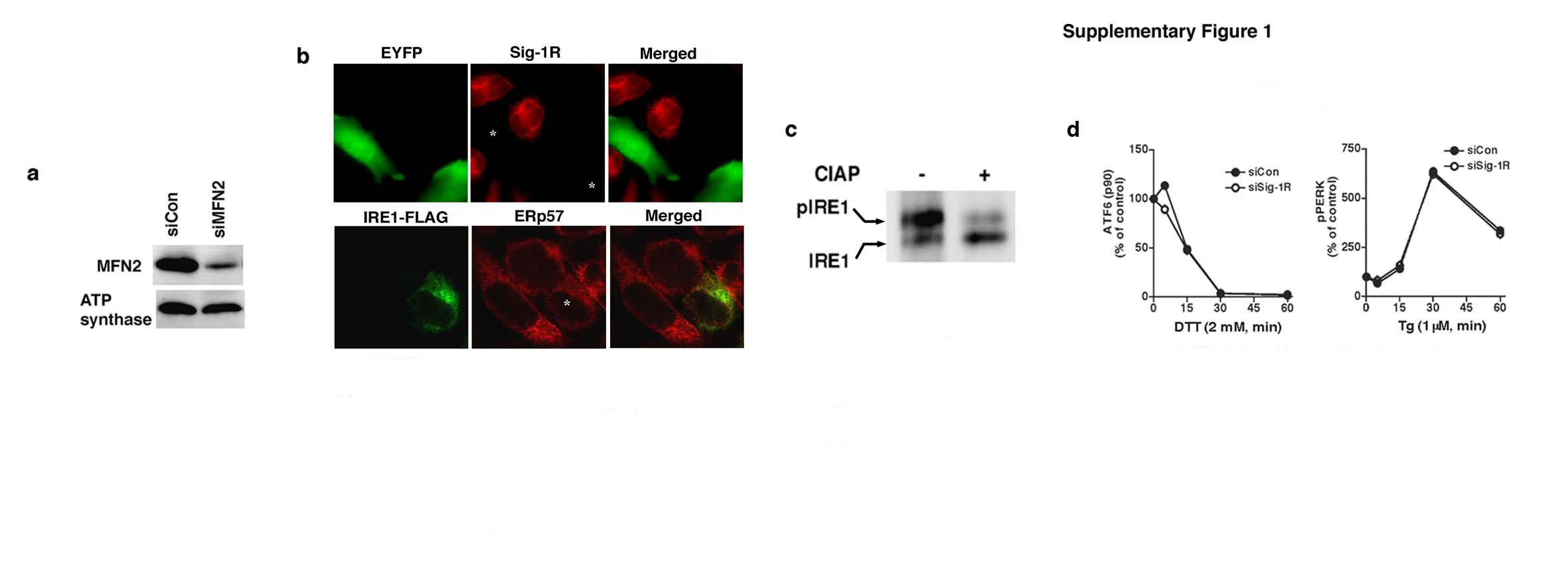

Supplement: Figure S1 — Sig-1R, mitofusin-2, and IRE1, ATF6 or PERK in CHO cells. (a) Knockdown of mitofusin-2 (MFN2) in CHO cells by siRNA. Scrambled (siCon) or MFN2 siRNA were trabsfected to CHO cells for 2 days. Protein levels were measured by immunoblotting. (b) Specific immunostaining of Sig-1Rs and IRE-1-FLAG in CHO cells. In top panels CHO cells transfected with siSig-1R accompanied by EYFP (asterisks) were immunostained with Sig-1R antibodies. Note that Sig-1R immunoreactivity was seen only in cells without transfection (no EYFP) confirming the specificity of Sig-1R staining. In bottom panels, transfected IRE1-FLAG (asterisks) and endogenous ERp57 were immunostained. Note: no FLAG immunoreactivity in non-transfected cells. (c) Effect of calf intestine alkaline phosphatase treatment (CIAP, 1 hr) on the phosphorylation status of IRE1. IRE1 in CHO cells were immunoprecipitated from cell lysates, and then tratened with CIAP for 1 hr before Western blotting. Note: CIAP decreases the upper band of IRE1 while concomitantly increases the lower band. (d) Effect of Sig-1R knockdown on the activation of ATF6 and PERK in CHO cells. DTT or thapsigargin was applied to activate those ER stress sensors. Activation of PERK was measured by pPERK immunostaining. Intensities of immunoblotted bands were measured and normalized to that of ERK. (TIF) [file pone.0076941.s001.tif]

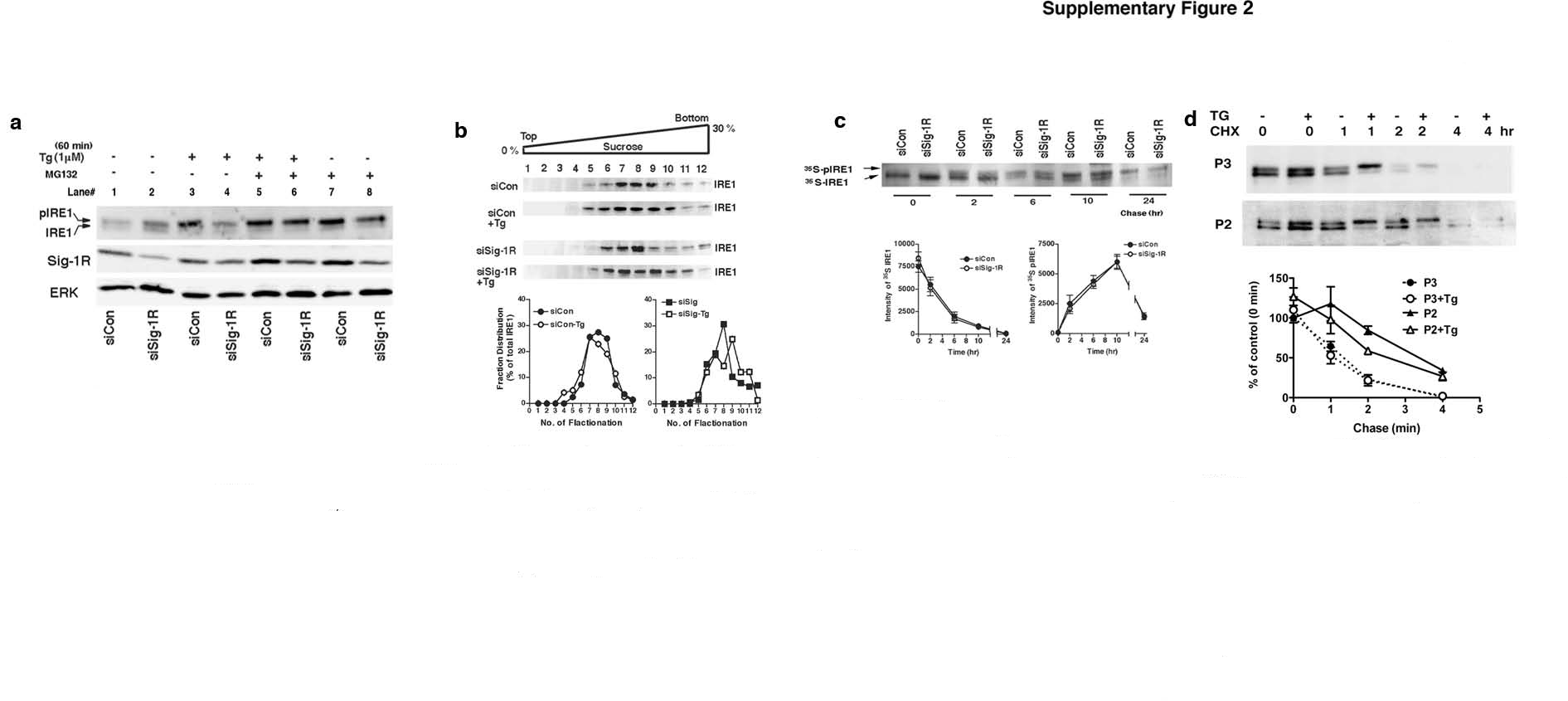

Supplement: Figure S2 — Effects of Sig-1R knockdown on the stability of IRE1. (a)Effects of MG132 (10 µM; applied 10 min before thapsigargin (Tg) on Tg (1 µM for 1 hr)-induced degradation of IRE1 in Sig-1R knockdown CHO cells. Note that the degradation of IRE1 caused by Sig-1R knockdown in Tg-treated cells (lane 4) was inhibited by MG132 (lane 8). (b) Sig-1R knockdown potentiates the aggregation of IRE1 induced by thapsigargin (Tg). siRNA-transfected CHO cells were stimulated with 1 µM of Tg for 1 hr in the presence of 10 µM of lactacystin (for 70 min). The total cell lysate was prepared with 1% Triton X-100 and subjected to sucrose gradient centrifugation. Twelve fractions were obtained from the top. Note the increase of IRE1 in high-density fractions 9–11 in Sig-1R knockdown cells treated with Tg. (c) Effect of Sig-1R knockdown on the stability of newly synthesized IRE1 in un-stressed CHO cells. CHO cells were pulse-labeled with 35S-methionine (S35-Met) for 10 min followed by chasing with excess cold methionine in the culture medium. IRE1 in S35-Met-labeled CHO cells were immunoprecipitated followed by autoradiography. Intensities of unphosphorylated IRE1 (left graph) and phosphorylated IRE1 (right graph) were densitometrically measured. Each point represents the means ±s.e.m. (n = 4). (d) Kinetics of IRE1 degradation at P3 (upper panel; the non-MAM ER microsomes) and P2 (bottom panel; crude mitochondrial fraction containing both MAM and mitochondrial fractions. After inhibition of protein synthesis by cycloheximide (5 mM, for 1 hr), Tg (1 µM) or vehicle (“−” sign in the panel) was applied to wild-type CHO cells. After the subcellular fractionation, levels of IRE1 were measured by immunoprecipitation of 20 µg of P2 proteins or 200 µg of P3 proteins. The graph represents the average of three independent experiments with s.e.m. Note that IRE1 at P3 fractions show a faster degradation when compared to the IRE1 at P2 fractions in both of the Tg(−) and Tg(+) samples. (TIF) [file pone.0076941.s002.tif]

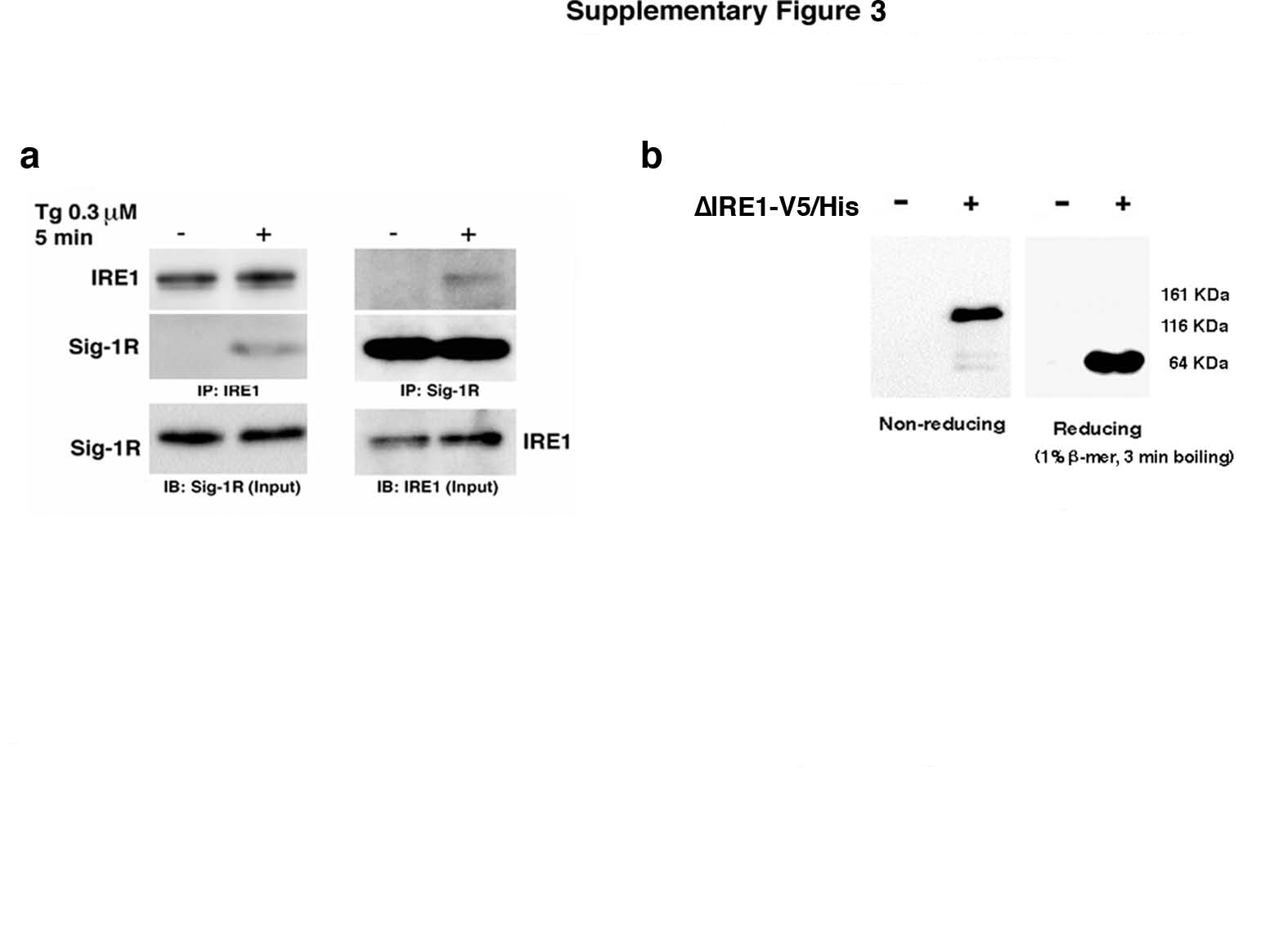

Supplement: Figure S3 — Association of Sig-1R with full-length IRE1 and the dimerization of IRE1. (a) ER stress-induced association of Sig-1Rs with IRE1. Before and after the treatment with thapsigargin (Tg), MAM fractions were prepared from CHO cells. Endogenous IRE1 or Sig-1R were immunoprecipitated. Co-immunoprecipitated counter proteins were measured by immunoblotting. Input: immunoblotting of total lysates. (b) Purified ΔIRE1-V5/His constitutively form dimmers under the non-reducing condition. ΔIRE1-V5/His was purified by a Ni+ column chromatography (see Methods). Lanes “−” represent the eluant from a Ni+ column pre-incubated with wild-type lysates (i.e., no expression of ΔIRE1-V5/His); lanes “+” represent the eluant from a column incubated with cell lysates containing overexpressed ΔIRE1-V5/His. Note that most of purified ΔIRE1-V5/His show the dimeric form in the non-reducing condition [i.e., without β-mercaptopethanol (β-mer)]. (TIF) [file pone.0076941.s003.tif]

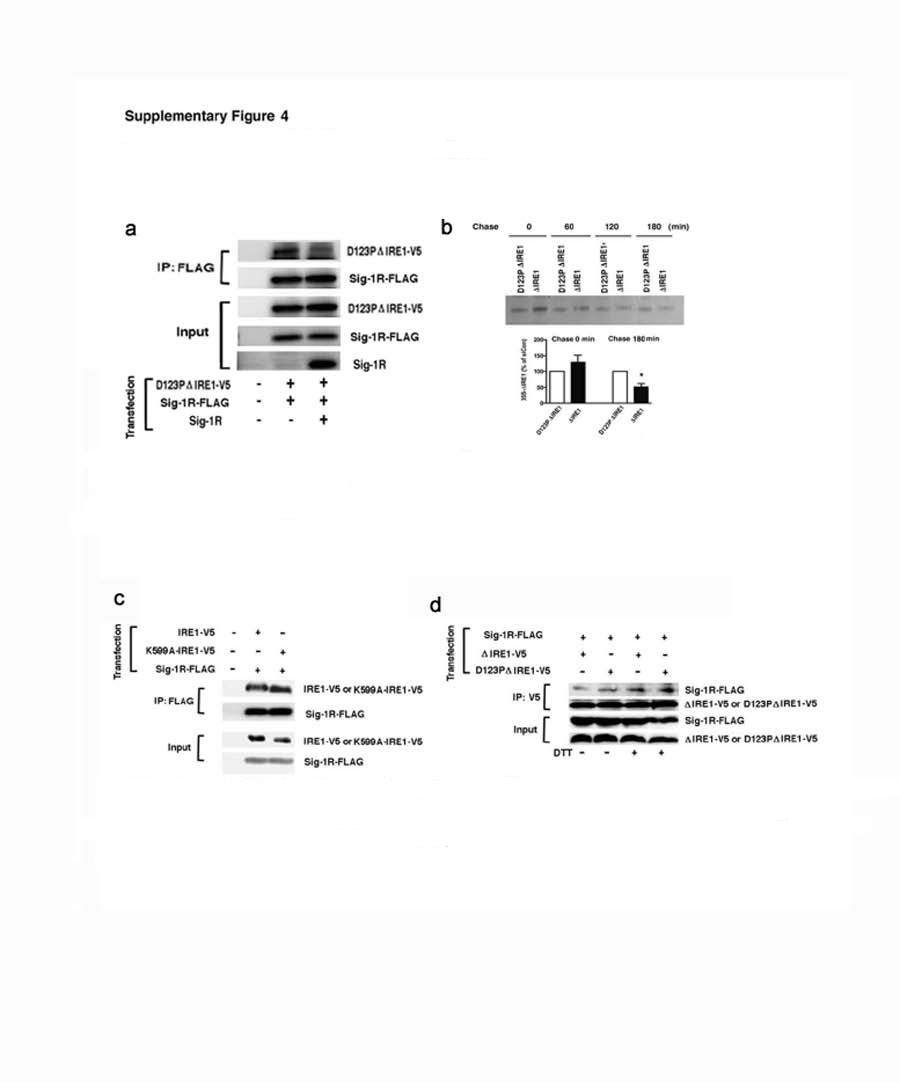

Supplement: Figure S4 — Domain interactions between Sig-1R and IRE1. (a) Evidence for the direct protein-protein interaction between Sig-1R and IRE1 via mass-action law by substituting tagged Sig-1R with overexpression of non-tagged Sig-1R. Effects of overexpressed non-tagged Sig-1Rs on the interaction between tagged Sig-1Rs and IRE1s. D123PΔIRE1 was co-immunoprecipitated with FLAG-tagged Sig-1Rs with or without overexpression of non-tagged Sig-1Rs. (b) The life-time of D123PΔIRE1 and ΔIRE1-V5. CHO cells were pulse-labeled with S35-methionine (35S-Met) for 10 min followed by chasing with excess cold methionine in the culture medium. The ΔIRE1-V5 in S35-Met-labeled CHO cells was immunoprecipitated with anti-V5 antibodies followed by detection by autoradiography. In the graph, band intensities of D123PΔIRE1-V5 at respective chase time were taken as 100% (means±s.e.m.; *p<0.05 by paired t-test, n = 3). (c) Association of Sig-1Rs with IRE1 does not depend on the phosphorylation status of IRE1. FLAG-tagged Sig-1Rs were immunoprecipitated. Co-immunoprecipitated V5-tagged IRE1 or mutant IRE1 (K588A-IRE1-V5) lacking the kinase activity was measured by immunoblotting. (d) Monomers of IRE1 induced by dithiothreitol (DTT) preferentially associate with Sig-1Rs. FLAG-tagged Sig-1Rs and V5-tagged ΔIRE1 (with/without mutation at D123) were expressed in CHO cells for co-immunoprecipitation. Note the increase of Sig-1R-FLAG co-immunoprecipitated with ΔIRE1-V5 by DTT treatment and/or D123 mutation. The DTT treatment did not affect the molecular weight or the level of Sig-1R-FLAG. (TIF) [file pone.0076941.s004.tif]
